# Shift in bacterial etiology from the CAPNETZ cohort in patients with community-acquired pneumonia: data over more than a decade

**DOI:** 10.1007/s15010-021-01605-w

**Published:** 2021-03-27

**Authors:** D. C. W. Braeken, A. Essig, M. Panning, R. Hoerster, M. Nawrocki, K. Dalhoff, N. Suttorp, T. Welte, M. W. Pletz, M. Witzenrath, G. G. U. Rohde, J. Rupp, for the CAPNETZ- Study Group, M. Dreher, C. Cornelissen, W. Knüppel, D. Stolz, N. Suttorp, W. Bauer, A. Mikolajewska, M. Witzenrath, W. Pankow, S. Gläser, D. Thiemig, M. Prediger, S. Schmager, M. Kolditz, B. Schulte-Hubbert, S. Langner, G. Rohde, C. Bellinghausen, M. Panning, C. Hoffmann, T. Welte, J. Freise, G. Barten, W. Kröner, M. Nawrocki, J. Naim, T. Illig, N. Klopp, M. Pletz, C. Kroegel, B. Schleenvoigt, C. Forstner, A. Moeser, D. Drömann, P. Parschke, K. Franzen, J. Rupp, N. Käding, M. Wouters, K. Walraven, D. Braeken, C. Spinner, A. Zaruchas, D. Heigener, I. Hering, W. Albrich, F. Waldeck, F. Rassouli, S. Baldesberger, S. Stenger, M. Wallner, H. Burgmann, L. Traby

**Affiliations:** 1 Department of Respiratory Medicine, Maastricht University Medical Centre (MUMC+), Maastricht, the Netherlands; 2 Institute of Medical Microbiology and Hygiene, University Hospital of Ulm, Ulm, Germany; 3 Institute of Virology, University Medical Center-University of Freiburg, Freiburg, Germany; 4 Medical Clinic III, Pulmonology, University Hospital Schleswig-Holstein, Lübeck, Germany; 5 CAPNETZ STIFTUNG, Hannover, Germany; 6 Department of Infectious Diseases and Pulmonary Medicine and Division of Pulmonary Inflammation, Charité-Universitätsmedizin Berlin, Berlin, Germany; 7 Department of Pneumology and German Center for Lung Research (DZL), Hannover Medical School, Hannover, Germany; 8 Biomedical Research in Endstage and Obstructive Lung Disease Hannover (BREATH), German Center for Lung Research (DZL), Hannover, Germany; 9 Institute for Infectious Diseases and Infection Control, Jena University Hospital, Jena, Germany; 10 Department of Respiratory Medicine, Medical Clinic I, Goethe University Hospital, Frankfurt/Main, Germany; 11 Department of Infectious Diseases and Microbiology, University Hospital of Schleswig-Holstein/Campus Lübeck, University of Lübeck, Ratzeburger Allee 160, 23538 Lübeck, Germany

**Keywords:** CAP, Pathogens, Culture, Diagnostics

## Abstract

To determine the most relevant pathogens for CAP in Germany, patients with radiologically confirmed pulmonary infiltrates and at least one clinical sign of lung infection were prospectively recruited within the CAPNETZ cohort from 2004 until 2016. In 990 out of 4.672 patients (21%) receiving complete diagnostics the most prominent change of pathogens was a decrease of S. pneumoniae (58% in 2004 to 37.5% in 2016; *p* ≤ 0.001, ρ =  − 0.148) and an increase of H. influenzae (12.2% to 20.8%; *p* = 0.001, ρ = 0.104).

## Brief report

To determine the most relevant pathogens for CAP in Germany, patients with radiologically confirmed pulmonary infiltrates and at least one clinical sign of lung infection were prospectively recruited within the CAPNETZ cohort from 2004 until 2016. In 990 out of 4.672 patients (21%) receiving complete diagnostics, the most prominent change of pathogens was a decrease of *S. pneumoniae* (58% in 2004 to 37.5% in 2016; p ≤ 0.001, *ρ* =  − 0.148) and an increase of *H. influenzae* (12.2% to 20.8%; *p *= 0.001, *ρ* = 0.104).

Community-acquired pneumonia (CAP) is one of the most frequent infectious diseases worldwide and associated with a high burden in mortality and morbidity, and adherence to treatment guidelines is associated with improved outcome. A major hallmark in setting up treatment guidelines in CAP is not only a profound knowledge about the bacterial etiology that may change over time on a pathogen and species level but also regarding resistance patterns. Acquisition of reliable data largely relies on the surveillance of bacterial culture from respiratory samples that is complemented by urine-antigen testing and molecular approaches when applicable.

In an early study from 1993 by Macfarlane et al., bacterial pathogens were detected in 44% of patients with lower respiratory tract infections, with *S. pneumoniae* and *H. influenzae* being the most frequent isolated bacteria in 67.4% and 17.4% of the cases [1]. Since then, a steady decline in the detection rates of *S. pneumoniae* has been documented worldwide, with a much stronger effect in the US than in Europe [2]. Studies trying to elaborate bacterial replacement scenarios in CAP are scarce and hampered by the fact that intensive bacterial testing in CAP patients is less frequently performed in clinical settings. Reasons are manifold, including difficulties in obtaining adequate respiratory materials from severely diseased and also older patients, but also cost issues in countries with capped diagnosis-related budgets. As an extreme, Alyacoubi et al. reported that complete microbiology testing for CAP was only performed in 2% of the patients at the European Gaza hospital [3]. In contrast, Uematsu et al. could show that guideline-concordant microbiology testing, including sputum tests, blood cultures and urine antigen tests conducted on the first day of hospitalization, was significantly associated with reduced 30-day mortality and increased likelihood of discharge [4]. But even in an optimized diagnostic setting when bacterial culture testing is combined with urine antigen testing for pneumococci and additional PCR-based testing from nasopharyngeal swabs are added, no pathogen was detected in 11- 44% of the cases [2].

Empirical treatment of CAP without iterative reflection of antimicrobial strategies by microbiological guidance is questionable, particularly in settings with a higher likelihood of resistance. Thus, in CAP patients with a high percentage of Gram-negative bacteria, e.g. older patients, antimicrobial treatment was found to be inappropriate in 16% of all microbiologically documented CAP cases [5].

According to major guidelines, culture diagnostic from respiratory samples of every inpatient, urinary antigen testing for Legionella and Pneumococcus, as well as blood cultures in moderate to severely impaired patients is seen as the gold-standard for guiding antimicrobial treatment. Based on this approach, we investigated changes in pathogen frequencies in 10.498 CAP patients from 2004 to 2016 within the multicenter observational CAPNETZ study. Inclusion criteria were age ≥ 18 years, new pulmonary infiltrate on chest X-ray, clinical symptoms of cough, purulent sputum, positive auscultation and/or fever. Patients were excluded if they had criteria for nosocomial pneumonia, had been hospitalized within the previous 28 days, if they were chronically immunosuppressed, human immunodeficiency virus infected or had active tuberculosis. Follow-up included a structured interview on outcome parameters, including death, at 30, 90 and 180 days. CRB-65 [**C**onfusion of new onset, defined as an abbreviated mental test score of ≤ 8, **R**espiratory rate of ≥ 30 breaths/min, **B**lood pressure ≤ 90 mmHg systolic or diastolic blood pressure ≤ 60 mmHg, **A**ge ≥ 65 years] scores were calculated based on the sum of points, with one point assigned for the presence of each criterion.

Patients were further categorized into two subgroups depending on the completeness of the diagnostic material for identifying a respiratory pathogen. Patients were attributed to the “complete diagnostics” group when urine-antigen testing for *S. pneumoniae/*Legionella *sp.* and at least one respiratory sample (sputum or BAL or tracheobronchial secretion) for conventional bacterial culture was obtained. No data on viral pathogens or non-cultivable bacteria were included in the analyses.

**Fig. 1:**
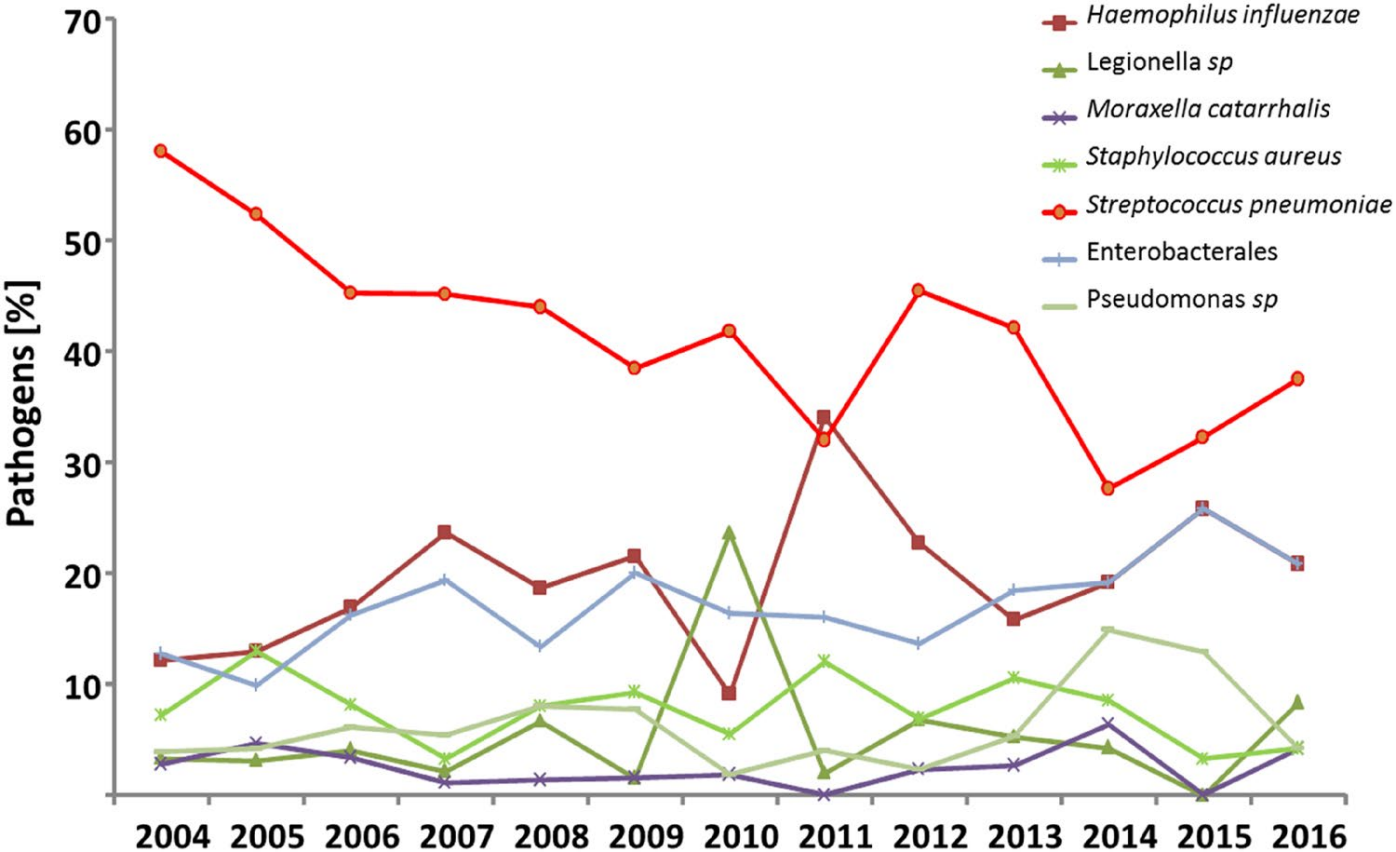
Distribution of bacterial pathogens detected in patients with complete diagnostics within the CAPNETZ cohort from 2004 to 2016. Association of pathogen detection with time was conducted using linear regression analysis by calculating Spearman's correlation coefficient (*ρ*)

In total, 10.498 patients were included in CAPNETZ from 2004 to 2016. When comparing patients with and without complete diagnostic material for pathogen detection (Table 1), patients with complete diagnostics were significantly younger (58.3 ± 17.6 vs. 62.2 ± 18.6 years; *p* ≤ 0.001) and more often female (58.2% vs. 54.5%; *p* ≤ 0.001). The groups did not differ regarding vaccination against the influenza virus but CAP-patients with complete diagnostics significantly more often obtained pneumococcal vaccination (13.4% vs. 11.6%; *p* ≤ 0.01). CAP severity was determined using CRB-65. Overall, patients with complete diagnostics were less severely ill showing significantly lower CRB-65 (0.8 ± 0.8 vs. 0.9 ± 0.9, *p* < 0.001) scores than patients with incomplete diagnostics. This was mainly because of an increase of patients presenting with a score of 0 which would not have required in-hospital treatment. A total of 4.672 patients were identified from 2004 to 2016 having complete diagnostic material for pathogen detection. Over the years, fewer patients were recruited to the study and in particular patients with complete diagnostics (650 in 2004 vs. 133 in 2016; *p* < 0.001, *ρ* =  − 0.038) significantly decreased. Among the bacterial pathogens detected, the most frequent pathogens were *Streptococcus pneumoniae*, *Haemophilus influenzae*, *Legionella* spp., *Staphylococcus aureus, Escherichia coli, Klebsiella* spp. and *Pseudomonas aeruginosa*. The most prominent changes in relative pathogen distribution that were observed during the study period were a decrease of *S. pneumoniae* from 58% in 2004 to 37.5% in 2016 (*p* ≤ 0.001, *ρ* =  − 0.148) and an increase of *H. influenzae* (*p* ≤ 0.001, *ρ* = 0.104) (Fig. 1). Clustering together pathogens belonging to the order Enterobacterales (“enteric Gram-negative bacilli”) an additional increase from 12.7% to 20.8% (*p* = 0.019, *ρ* = 0.075) was observed, while detection rates remained stable for Pseudomonas *sp*. (3.9% in 2004 vs. 4.2% in 2016) with only minor deviations (Fig. 1).

**Table 1:** Characteristics of CAP patients with complete and incomplete diagnostics

	Complete diagnostics (*n* = 4672)	Incomplete diagnostics (*n* = 5826)	*p* value
Age	58.3 ± 17.6	62.2 ± 18.6	< 0.001
Gender, female	1951 (58.2)	2651 (54.5)	< 0.001
Vaccination			
Influenza < 12 month (*n* = 4577/5473)	1578 (34.9)	1949 (36.0)	ns
Pneumococci (*n* = 4555/5439)	602 (13.4)	625 (11.6)	< 0.01
Hospitalised	2964 (63.4)	4516 (77.5)	< 0.001
Length of stay (*n* = 3013/4573)	10.0 (7.0–14.0)	10.0 (7.0–13.0)	ns
ICU (*n* = 1881/2831)	156 (8.5)	268 (9.7)	ns
CRB-65 (*n* = 4290/5419)	0.8 ± 0.8	0.9 ± 0.9	< 0.001
0	1806 (42.5)	1937 (36.1)	< 0.001
1	1771 (41.7)	2171 (40.5)	
2	570 (13.4)	999 (18.6)	
3	90 (2.1)	226 (4.2)	
4	10 (0.2)	30 (0.6)	

Variables were tested for normality using the Kolmogorov–Smirnov and Shapiro–Wilk test. Continuous data are presented as mean ± standard deviation (SD) or median (interquartile range), as appropriate, and categorical data as counts (relative percentages). To compare baseline characteristics and CAP severity rates between patients with and without complete diagnostics, independent samples *T* test, Mann–Whitney *U* test and Chi-square test were performed, as appropriate

*CAP* Community-acquired pneumonia, *CRB-65* confusion, respiratory rate ≥ 30/min, blood pressure (systolic < 90 mmHg or diastolic ≤ 60 mmHg), age ≥ 65, *ICU* intensive care unit

This is the first study showing the distribution of respiratory bacterial pathogens in CAP patients over more than a decade in a multicenter observational study in Germany. From 2004 to 2016 not only frequencies of patients obtaining complete microbiological diagnostics decreased but also pathogen detection significantly declined in patients with complete (*p* ≤ 0.001, ρ =  − 0.060) and incomplete diagnostics (*p* = 0.017, ρ = -0.031). This could be interpreted as a matter of reduced adherence to the study protocol but also reflects current practices in daily routine. As expected, detection rates were significantly higher in the patient group with complete diagnostics (21.2% vs. 6.4%, *p* ≤ 0.001), showing a significant decrease in the detection of *S. pneumoniae* and a significant increase in the detection of *H. influenzae* and Enterobacterales from 2004 to 2016. Importantly, the numbers of patients that were pre-treated with an antibiotic within the last 4 weeks before inclusion into the study did not change over the years (*p* = 0.161, *ρ* = -0,021).

In previous studies when relying completely on cultivation from respiratory samples, a bacterial pathogen could just be detected in one out of five CAP patients. Thus, in a study by Ieven et al., *S. pneumoniae* and *H. influenzae* were detected in 9.2% and 14.2% of the samples of CAP patients, respectively [6]. Introducing molecular testing results in higher numbers of detected pathogens (87% vs. 39% for culture-based methods) including viruses in 30% of the cases and frequently in combination with a bacterial pathogen [7]. Albeit the causative role of different viruses is still discussed controversially, in patients with a low CURB-65 score viruses were attributed to 5–29% of the cases, not requiring antimicrobial treatment [2]. With respect to the detection of bacterial pathogens, the likelihood of cultivating a bacterial pathogen from respiratory samples is supposed to be more successful in more advanced diseases and purulent sputum production [8]. However, this might be contradicted by the fact that in more severe diseases the collection of sputum is hampered by the clinical state of the patient.

A general decrease in *S. pneumoniae* over the last decades has been observed with a much stronger effect in the USA than in Europe [2]. This decrease has been associated with herd protection effects due to PCV7 (introduced in 2007), which was replaced by PCV13 in 2010 [9], but does not explain the decline in *S. pneumoniae* detection rates observed in our study before 2007. Gram-negative bacilli, *Staphylococcus aureus* and atypical bacteria were each identified in 2–5% of patients that required hospitalization, not showing a significant trend in the replacement of *S. pneumoniae* [2]. In Italy, replacement of *S. pneumoniae* by non-typeable *H. influenzae* (NTHI) has been shown after vaccination with PCV13, arguing for a colonization competition between these two pathogens as part of the colonizing flora at least in children [10]. Furthermore, preceding PPV23 vaccination was an independent risk factor for *H. influenzae* CAP, suggesting also replacement [11]. In a recent study from Vestjens et al., the proportion of pneumococcal CAP significantly decreased from 37 to 26% comparing the pre-PCV7 period in 2004 with the PCV10 period in 2016. However, no significant sustained shifts in the relative contribution of other bacteria to the aetiology of CAP were observed [12]. In microbiological settings with a high proportion of urine-antigen tests for the detection of *S. pneumoniae* infections, it has to be considered that e.g. the sensitivity of the BinoxNow urine- antigen test for *S. pneumoniae* decreased due to a PCV-13 caused shift from serotypes 9V, 14, 18C and 20, showing high sensitivity in the test, to the serotypes 23B, 9L/N, 11A and 8, showing lower test sensitivities [13]. In particular, the percentage of serotype 3 continuously increased despite the introduction of PCV13 in 2010 [14], which could account at least in part for the reduced detection of *S. pneumoniae* by conventional urine-antigen testing within the study.

There are some limitations of the study that need to be acknowledged. Due to the declining number of patients with complete microbiological diagnostics over the observed period of time the total annual numbers of bacterial isolates for the statistical analysis were small for the last years of the observation period. The reasons, therefore, are manifold and cannot only be attributed to the recommendations for testing only critically ill patients within the last decade. In addition, testing for *Mycoplasma pneumoniae* is missing in this analysis as it was only performed in 2011–2012 with a frequency of 12.3% [15]. Furthermore, a substantial proportion of patients were pre-treated with antibiotics, thus decreasing the diagnostic yield [16].

Taken together, it is important to note that pathogen detection rates were significantly higher in patients that obtained complete diagnostics. Incorporation of quantitative PCR- testing may further increase the overall detection rates for the most frequent bacteria, and also enhance the recognition of viral co-infections that are independent risk factors for disease severity [17, 18]. One caveat that has to be considered in the PCR-based multiplex-testing for CAP is that Enterobacterales are currently not included in the different panels and that the increased sensitivity in pathogen detection has to be clinically validated, first. Further efforts should be undertaken in the clinical setting for patients with moderate to severe CAP identifying a causative pathogen of the disease. Not only for adjusting individual treatment decisions but also to modify empirical treatment strategies in future guidelines if the current trend showing a replacement of *S. pneumoniae* by *Haemophilus influenzae* and Enterobacterales further evolves.
